# Synthesis of anisotropic Janus composite particles based on polystyrene/urushiol- lanthanum chelate polymer

**DOI:** 10.1371/journal.pone.0314449

**Published:** 2024-12-03

**Authors:** Junhui He, Yi Zhang, Shuyan Li

**Affiliations:** 1 College of Petrochemical Engineering, Zhangzhou Institute of Technology, Zhangzhou, China; 2 Fujian Collaborative Innovation Center of Fine Chemicals, Zhangzhou, Fujian, China; Brandeis University, UNITED STATES OF AMERICA

## Abstract

To expand the potential applications of raw lacquer, snowman-like polystyrene (PS)-urushiol lanthanum (ULa) Janus composite particles were synthesized by emulsion swelling-assisted protrusion from PS/ULa core-shell composite microspheres. The morphology and chemical composition of the PS/ULa composite microspheres and the PS-ULa Janus composite particles were investigated with scanning electron microscopy (SEM), transmission electron microscopy (TEM), energy dispersive X-ray (EDX), thermogravimetric analysis (TGA), and Fourier transform infrared (FT-IR). The PS-ULa Janus particles were compartmentalized into two parts, each with a different morphology and chemical composition. Results showed that the intact ULa shell with appropriate thickness is a crucial factor for controllable swelling, and the thickness of the PS/ULa core-shell composite microsphere could be controlled by polymerization temperature. This anisotropic Janus particle exhibits potential applications in orienting materials, such as directional catalysis.

## Introduction

Raw lacquer is a renewable and eco-friendly biopolymer resource that has garnered significant research attention due to its remarkable properties, including high thermal stability, superior durability, and antimicrobial characteristics [1–4]. Historically, much of the research on raw lacquer has focused on its applications as a natural coating and high-quality natural paint. Urushiol, the principal component of raw lacquer, is a catechol derivative characterized by a long unsaturated hydrocarbon side chain [5]. The presence of two adjacent phenolic hydroxyl groups in urushiol enables it to react efficiently with metal compounds, forming urushiol-metal compounds [6–8]. Notably, urushiol-rare earth chelate polymers exhibit excellent catalytic performance and thermal stability [9,10].

Janus particles have garnered significant attention in recent years due to their unique structures and properties, exhibiting great potential in various applications, including biomedical science [11], functional coatings [12,13], and catalysts [14]. Numerous strategies for preparing Janus composite particles have been developed, including the Pickering emulsion method [15], self-assembly [16], directional UV-induced reactions [17], and plasma etching strategies [18]. Among these, the emulsion swelling-assisted protrusion method [19] offers a mass and controllable approach for preparing Janus particles, effectively addressing the challenges associated with their development. Additionally, the use of lacquer induction in the preparation of Janus particles holds significant promise for expanding the applications of raw lacquer, with the potential for preparing anisotropic organic polymer functional materials.

The Janus composite particles of urushiol-lanthanum chelate-polystyrene (PS-ULa) were synthesized through the emulsion swelling-assisted protrusion of PS/ULa core-shell composite microspheres. The morphology and chemical composition of both PS/ULa core-shell composite microspheres and PS-ULa Janus composite particles were analyzed and discussed in detail. Furthermore, we focused on the effects of polymerization temperature on the shell structure of the PS/ULa core-shell microspheres and their swelling process. This anisotropic Janus particle exhibits potential applications in orienting materials, such as directional catalysis.

## Experimental

### Materials

Chinese lacquer was offered by the Institute of Lacquer, Xi’an, China. Urushiol was extracted from raw lacquer with acetone. Styrene (St), ethanol, concentrated sulfuric acid, Hydrochloric acid, Lanthanum oxide (La_2_O_3_), and sodium dodecyl benzene sulfonate (SDBS) were purchased from Sinopharm Chemical Reagent Co., Ltd. 1,4-dimethylbenzene (p-xylene) was purchased from Tianjin Fuchen Chemical Reagents Factory, all chemicals were of analytical grade. St was purified with alkaline Al_2_O_3_ to remove the inhibitor. The anhydrous lanthanum chloride (LaCl_3_) was prepared according to the reported literature [20].

### Fabrication of PS/ULa core-shell composite microspheres

The sulfonated polystyrene (SPS) microspheres were prepared according to the literature [6]. After mixing and dispersing the saturated ethanol solution of LaCl_3_ and SPS microspheres, stir them at room temperature for more than 10 h. It was dispersed and centrifuged several times with ethanol to remove excess La^3+^, then added 1% (wt) solution of urushiol dispersed in ethanol, followed by stirring at various polymerization temperatures, including room temperature, 50°C, and 70°C for 24 h, respectively. Then, The PS/ULa core-shell composite microspheres were obtained after centrifuging and washing with ethanol.

### Fabrication of PS-ULa Janus composite particles

The PS/ULa core-shell composite microspheres and the appropriate amount of p-xylene solvent were emulsified in 1% (wt) SDBS aqueous solution under ultrasonication, respectively. Then, a certain amount of p-xylene emulsion was added to the dispersion of PS/ULa core-shell composite microspheres under stirring at room temperature for 20 min. The PS-ULa Janus composite particles were achieved by washing and drying.

### Characterization

The morphology of the samples was characterized using a JSM-7500F scanning electron microscope (SEM) operating at a voltage of 5 kV. The chemical composition was analyzed with an Oxford Inca Energy-dispersive X-ray (EDX) system, which was employed in conjunction with the SEM. Transmission electron microscopy (TEM) images were acquired using a JEM-2010 electron microscope. The dried samples were analyzed using the KBr pellet method on a Nicolet-5700 Fourier transform infrared (FT-IR) spectrometer. Thermal gravimetric analysis (TGA) was performed with a METTLER TGA/SDTA 851 at a heating rate of 10°C/min under a nitrogen flow.

## Results and discussion

### Microscopic morphology of PS/ULa core-shell composite microspheres and PS-ULa Janus composite particles

**Fig 1:**
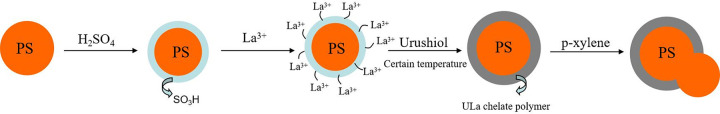
Schematic diagram for the preparation of PS-ULa Janus composite particles.

The schematic diagram for the preparation of PS-ULa Janus composite particles was presented in Fig 1. Initially, SPS microspheres were synthesized by sulfonating PS microspheres with concentrated sulfuric acid. Subsequently, the surface of the SPS microspheresis was covalently bonded with a high density of sulfonic groups, which reacted with La^3+^ through an ion exchange. The 4f empty orbital of La^3+^ retained its capacity to accept electrons. Following this, La^3+^ coordinated with two adjacent phenolic hydroxyls groups from urushiol and form a ULa chelate compound. An oxidative polymerization reaction occurred between the unsaturated side chains of urushiol under the catalysis of lanthanum element, resulting in the formation of PS/ULa core-shell composite microspheres. Finally, the PS/ULa core-shell composite microspheres were swollen in p-xylene solvent. Due to the pressure differential between the ULa shell and the PS core, the PS core experiences rupture and protrusion, resulting in the formation of snowman-like PS-ULa Janus composite particle.

**Fig 2:**
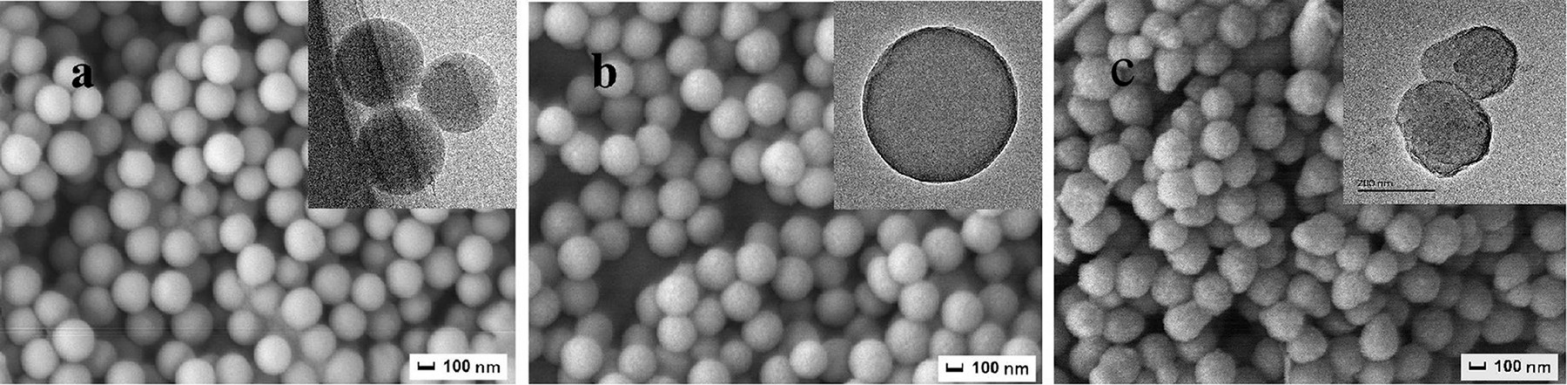
SEM and TEM images. (a) PS, (b)PS/ULa core-shell composite microsphere, (C) PS-ULa Janus composite particle: PS/ULa core-shell composite microsphere were polymerized at 70°C.

Fig 2 showed the SEM and TEM images of PS microsphere, PS/ULa core-shell composite microsphere and PS-ULa Janus composite particle. In comparison to the PS microspheres (Fig 2A), the surface of PS/ULa exhibited increased roughness, with the average diameter rising from 250 nm to 256 nm (Fig 2B). The TEM image confirmed the presence of a distinct core-shell structure in the PS/ULa particles. These observations collectively indicate the successful synthesis of the PS/ULa core-shell composite microspheres. Fig 2C illustrates the asymmetric, snowman-like Janus composite particles of PS-ULa, derived from the swelling of PS/ULa core-shell composite microspheres in p-xylene solvent. Notably, the two ends of the PS-ULa Janus composite particles exhibit distinct morphologies; the ULa knob features a rough surface and a larger average particle size, allowing for clear differentiation between the two ends of the PS-ULa Janus composite particles.

### Chemical composition of PS-ULa core-shell composite microspheres and compartmentalization of PS-ULa Janus composite particles

**Fig 3:**
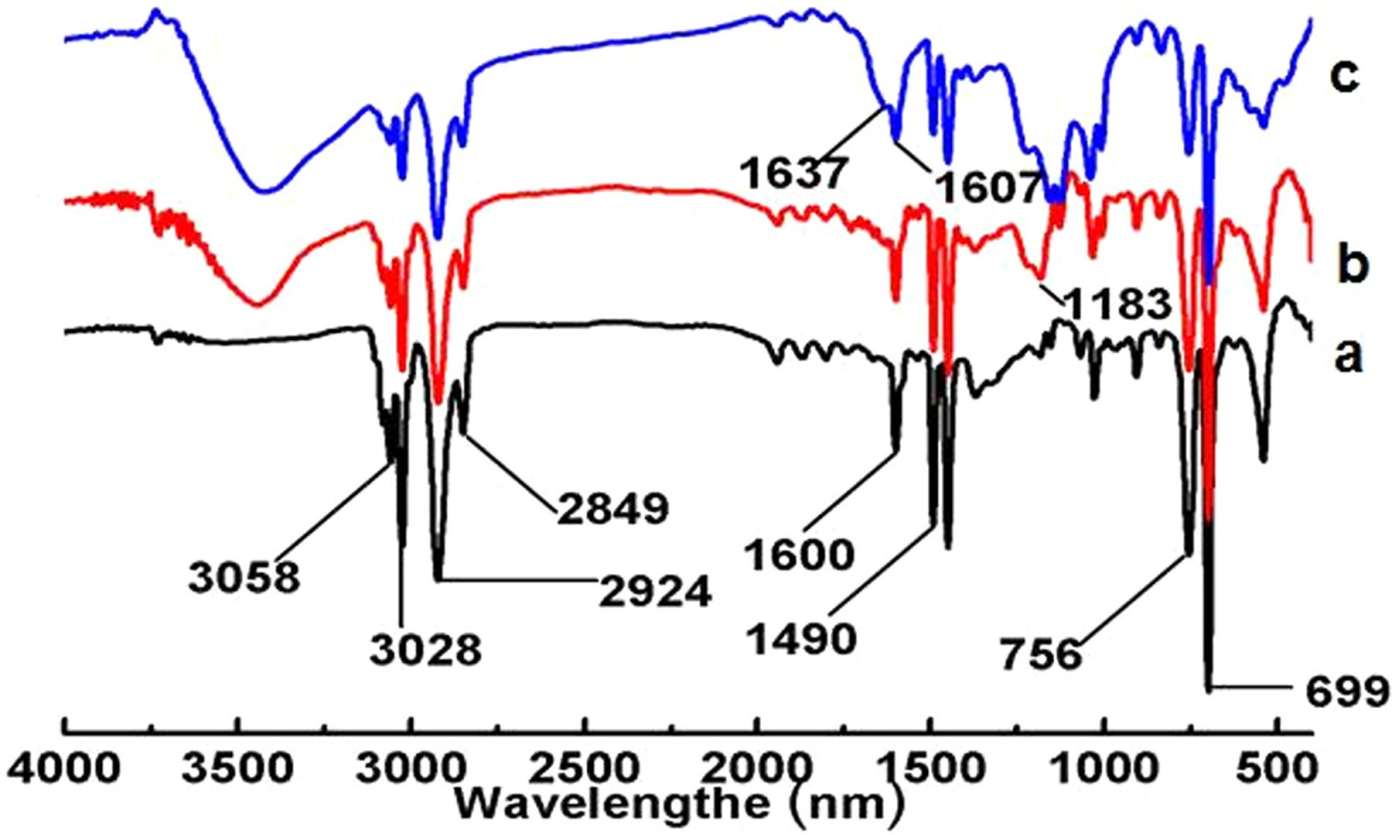
FT-IR spectra. (a) PS, (b) SPS microsphere, (c) PS/ULa core-shell composite microsphere.

Fig 3 illustrated the FT-IR spectra of PS (Fig 3A), SPS (Fig 3B), and PS-ULa core-shell composite microspheres (Fig 3C). The spectrum of PS displays characteristic absorption bands at 2924 and 2849 cm⁻^1^, corresponding to the stretching vibrations of -CH₂ and -CH, as well as at 1600 and 1490 cm⁻^1^, which are associated with the C = C stretching of benzene rings. Additionally, absorption peaks at 758 and 699 cm⁻^1^ are observed, representing the out-of-plane bending of C-H in mono-substituted benzene rings. The peak at 1183 cm⁻^1^ is attributed to the -SO₃H group, confirming the successful synthesis of SPS. Notably, the distinct absorption peaks at 1637 and 1601 cm⁻^1^ are characteristic of the benzene ring in urushiol. Furthermore, the disappearance of the peak at 1183 cm⁻^1^ serves as additional evidence for the reaction between La^3^⁺ and SPS microspheres.

**Fig 4:**
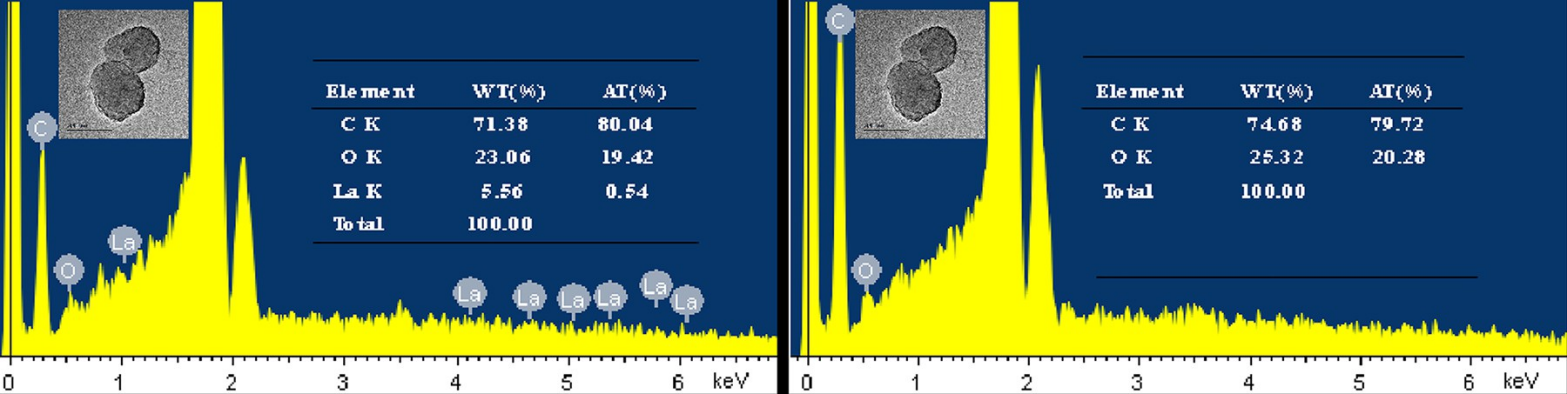
EDX spectra of the PS-ULa. (a) ULa knob, (b) PS knob.

The EDX analysis (Fig 4) revealed distinct compositions in the two knobs of PS-ULa. Specifically, the ULa knob exhibited the presence of the lanthanum element, whereas the PS knob contained only carbon (C) and oxygen (O) elements. The combined results from FT-IR and EDX confirm the successful synthesis of Janus composite particles featuring anisotropic PS-ULa.

**Fig 5:**
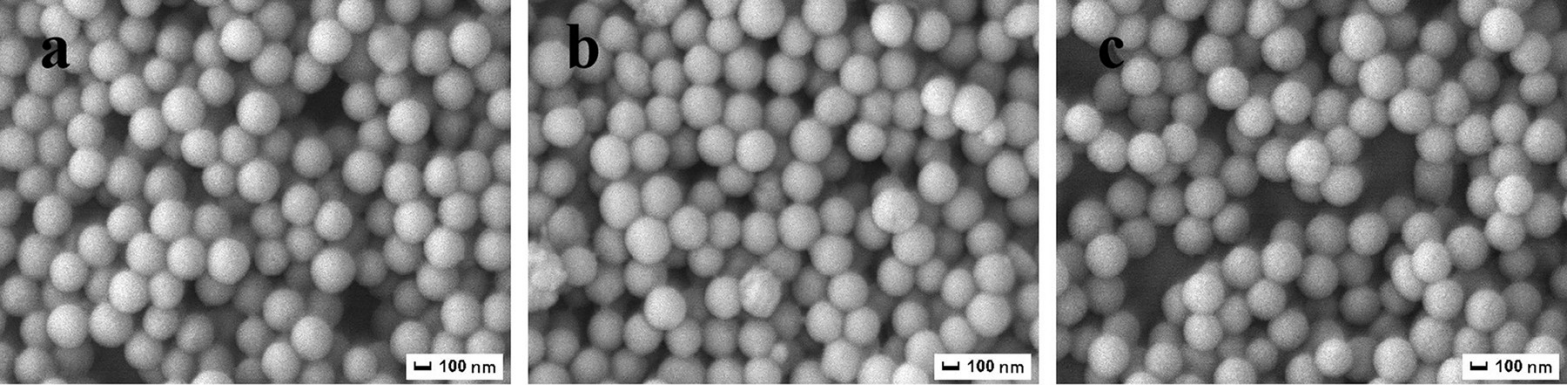
SEM images of PS/ULa core-shell composite microsphere polymerized at (a) room temperature, (b) 50°C, (c) 70°C.

Fig 5 presented SEM images of PS/ULa core-shell composite microspheres that were polymerized at various temperatures, including room temperature, 50°C, and 70°C. It is evident that as the polymerization temperature increases, the surface roughness of the PS/ULa also escalates. Correspondingly, the average particle size rises from 250 to 252 and 253 nm, respectively. These findings suggest that the thickness of the shell layer in PS/ULa can be regulated by adjusting the polymerization temperature.

**Fig 6:**
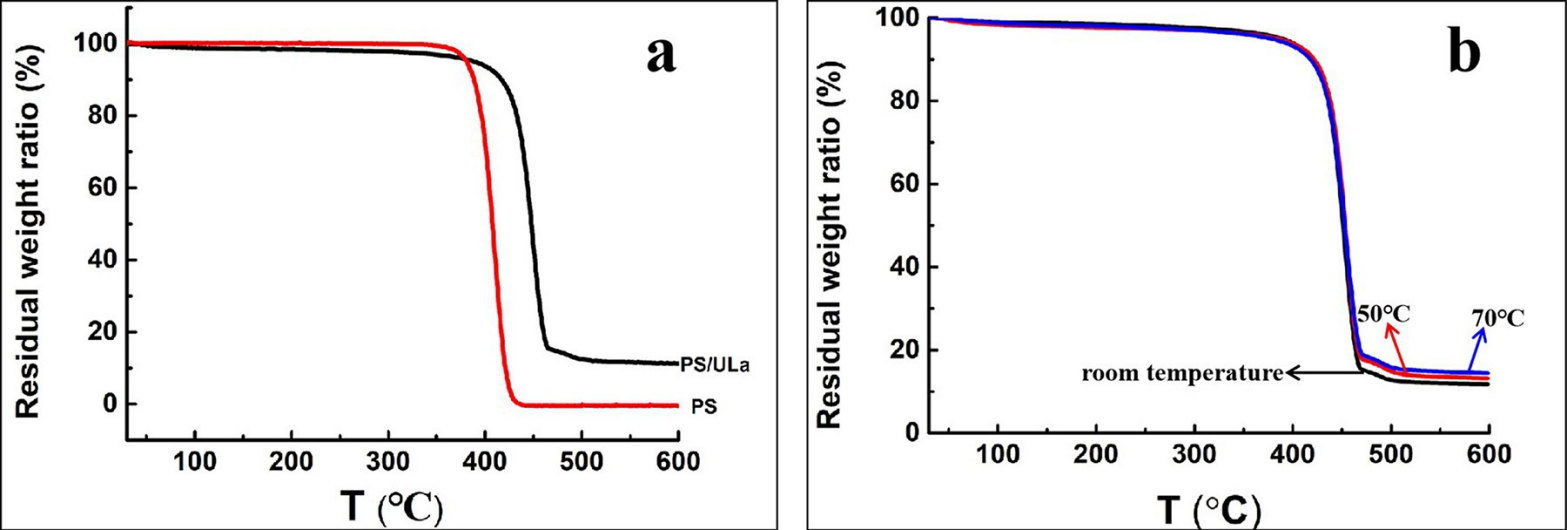
TG curves of (a) PS microspheres and PS/ULa core-shell composite microspheres polymerized at 70°C, (b) PS/ULa core-shell composite microsphere polymerized at room temperature, 50°C and 70°C.

Fig 6 showed the TGA curves of PS microspheres and PS/ULa core-shell composite microspheres polymerized at varying temperatures. As illustrated in Fig 6A, the thermal degradation of pure PS occurs at 408°C, whereas the PS core within the PS/ULa core-shell composite microspheres exhibits a degradation temperature of 449°C. This significant increase in thermal degradation temperature for the PS core in the core-shell microspheres can primarily be attributed to the urushiol lanthanum polymer coating on the shell, which enhances the thermal stability of the PS core. As shown in Fig 6B, despite variations in polymerization temperatures for the core-shell microspheres, their thermal behavior patterns remain consistent. Notably, there is an increase in residual weight ratio from 11.8% (room temperature) and 13.2% (50°C) to 14.4% (70°C) attributing to the moderate thermal crosslinking of urushiol [21].

**Fig 7:**
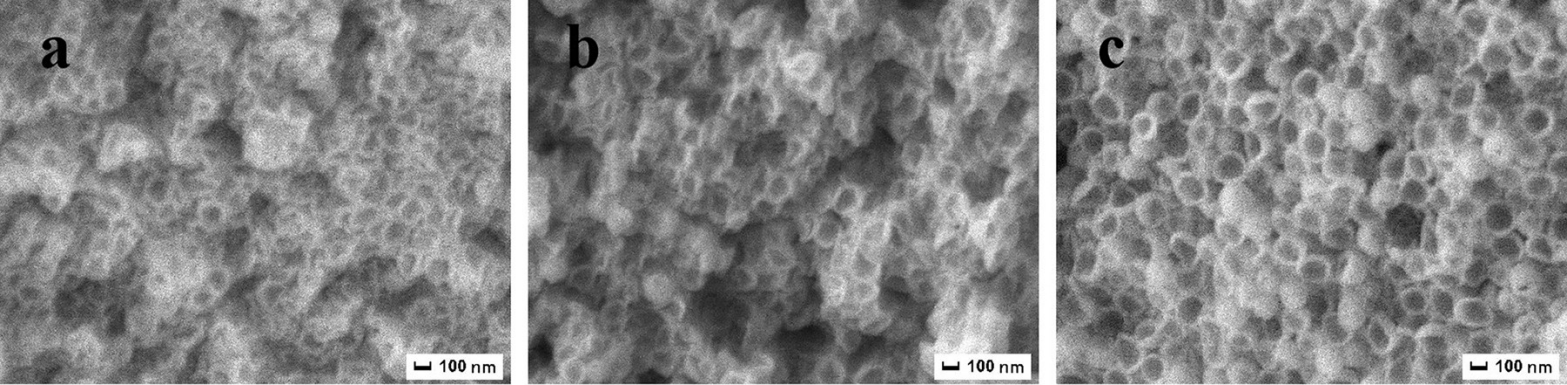
SEM images of sintered PS/ULa core-shell composite microspheres polymerized at (a) room temperature, (b) 50°C, (c) 70°C.

Fig 7 illustrated the SEM images of PS/ULa composite microspheres after sintering. During the sintering of PS/ULa core-shell composite microspheres, the PS core initially undergoes decomposition, generating a relatively high internal pressure that causes the ULa shell to split into two halves, resulting in a bowl-shaped structure. The figure showed increased polymerization temperature correlates with a more complete and thicker PS/ULa composite shell layer. The analytical results further provide compelling evidence that modifying the polymerization temperature can significantly influence the thickness of the shell layer within PS/ULa core-shell composite microspheres.

### Effect of polymerization temperature on the swelling behavior of PS-ULa Janus composite particles

**Fig 8:**
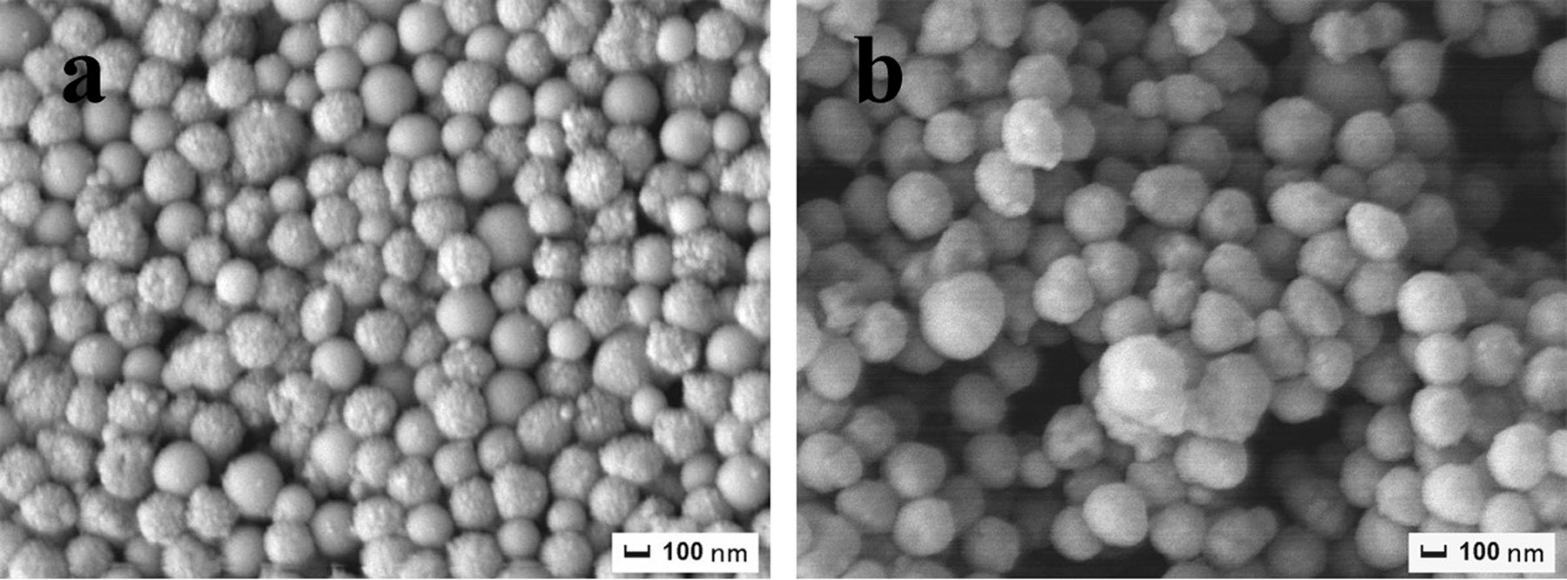
SEM images of PS-ULa Janus composite particles. PS/ULa core-shell composite microspheres were polymerized at (a) room temperature, (b) 50°C.

Figs 2C and 8 present the SEM images of PS-ULa Janus composite particles, which were synthesized through the swelling of the xylene monomer. Notably, in comparison to the swelling of PS/ULa core-shell composite microspheres synthesized at room temperature (Fig 8B) and 50°C (Fig 8C), the ULa end of the snowman-shaped Janus composite particles polymerized at 70°C (Fig 2C) exhibits a significantly fuller appearance. This observation can be attributed to the fact that during polymerization at 70°C, the side chain unsaturated hydrocarbons underwent complete oxidation and polymerization, resulting in a thick and intact shell. During swelling in xylene, this robust shell effectively resisted the initial increase in internal osmotic pressure. However, once this pressure reached a certain threshold, internal PS protruded from the weakest point within the shell. In contrast, shells polymerized at room temperature and 50°C are relatively softer and thinner. During their swelling process, reduced internal support forces lead to an inability to maintain structural integrity, which results in issues such as soft collapse or insufficient protrusion.

## Conclusions

PS-ULa Janus composite particles containing urushiol and lanthanum were successfully synthesized using an emulsion swelling-assisted protrusion method from PS/ULa core-shell composite microspheres. To our knowledge, achieving the appropriate thickness of the ULa shell is a critical factor in the swelling process. In this study, the thickness of the shell layer of PS/ULa core-shell composite microspheres was controlled by varying the polymerization temperature. As the polymerization temperature increased from room temperature to 70°C, the oxidative polymerization of unsaturated hydrocarbons became more complete and efficient. Consequently, the PS/ULa core-shell composite microspheres were swollen with xylene monomer, leading to the protrusion of the PS core and the formation of PS-ULa Janus composite particles. Compared to the ULa shell polymerized at room temperature and 50°C, the polymerization at 70°C provided a stronger external support force for the core-shell structure during the swelling process, resulting in more complete snowman-like Janus microspheres. This anisotropic Janus particle exhibits potential applications in orienting materials, such as directional catalysis.
